# 3D reconstruction of laser projective point with projection invariant generated from five points on 2D target

**DOI:** 10.1038/s41598-017-07410-6

**Published:** 2017-08-01

**Authors:** Guan Xu, Jing Yuan, Xiaotao Li, Jian Su

**Affiliations:** 1 Traffic and Transportation College, Nanling Campus, Jilin University, Renmin Str. 5988#, Changchun, China; 2 School of Mechanical Science and Engineering, Nanling Campus, Jilin University, Renmin Str. 5988#, Changchun, China

## Abstract

Vision measurement on the basis of structured light plays a significant role in the optical inspection research. The 2D target fixed with a line laser projector is designed to realize the transformations among the world coordinate system, the camera coordinate system and the image coordinate system. The laser projective point and five non-collinear points that are randomly selected from the target are adopted to construct a projection invariant. The closed form solutions of the 3D laser points are solved by the homogeneous linear equations generated from the projection invariants. The optimization function is created by the parameterized re-projection errors of the laser points and the target points in the image coordinate system. Furthermore, the nonlinear optimization solutions of the world coordinates of the projection points, the camera parameters and the lens distortion coefficients are contributed by minimizing the optimization function. The accuracy of the 3D reconstruction is evaluated by comparing the displacements of the reconstructed laser points with the actual displacements. The effects of the image quantity, the lens distortion and the noises are investigated in the experiments, which demonstrate that the reconstruction approach is effective to contribute the accurate test in the measurement system.

## Introduction

In recent years, 3D reconstruction based on camera has attracted wide attention of researchers and has been applied in various research fields. E.g. Mian^1^ extracts features from the different angular face images that are acquired by using the computer screen as a kind of coded illumination and reconstructs the 3D profile models by using a new efficient algorithm. Kim^2^ presents a linear layered method including the affine recovery and the metric recovery. The camera calibration and 3D reconstruction are performed by utilizing the scene geometry.

Vision measurement on the basis of structured light is a very significant way of reconstructing 3D object surfaces due to non-contact test, simple operation, high efficiency and good accuracy^3–6^. Nguyen^7^ presents a real-time measurement method of the object shape with the advantages of high speed and accuracy. A multithread parallel algorithm is adopted to deal with the acquired images by a camera and a programmable projector. A process is provided by Qin^8^ to detect the terrestrial change at street level by combining point clouds and terrestrial pictures. It inspects the consistency of point clouds and stereo images by rectifying and re-projecting stereo portions of the terrestrial pictures. A method using structured light for the 3D shape reconstruction is proposed by Dipanda^9^ for automatically obtaining information of the objects. The correspondence procedure in real-time is realized by the cell algorithm. Xu^10^ describes the solution method of the planar structured light in the vision-based inspection by employing the Plücker matrices that shows a precise calibration of the planar structured light under the impact factors of the image quantity and the noise. Saeed^11^ introduces a method of extracting the weld pool surface information from the images. The calibrated charge-coupled sensor is used to capture the mirrored laser beam to constitute the images. The depth of the weld pool surface can be calculated by utilizing the information of the charge-coupled device sensor and the location of the laser. Chen^12^ proposes a method to reconstruct the surface of a wheel with the data from the vision sensor. The wheel surfaces are obtained from two structured-light-sensors which are calibrated by the iteration of the closest point. Li^13^ outlines a method to improve the Fourier transform profilometry (FTP). According to the geometrical conditions, the map of the light phase is recovered by a gray-scale image of the fringe. Yun^14^ presents a framework to improve the FTP method by retrieving the absolute phase pixel-by-pixel. Two images with different frequencies are adopted in the approach. The high-frequency phase is generated from the low-frequency phase. An accurate and effective camera calibration technology is particularly important for the 3D reconstructions of objects. Three kinds of the calibration systems for the camera are recorded in the previous works: the 3D calibration system^15–17^, the 2D planar calibration system^18–21^ and the 1D linear calibration system^22–24^. 3D calibration system obtains the parameters of a camera by only one captured image. Meanwhile, high accuracy is achieved by the 3D calibration target. However, in order to extract enough feature points, the 3D target needs to be manufactured to a certain volume and the three faces of the volume must be accurately vertical to each other. Therefore, the process and transportation of the 3D target are time-consuming and inconvenient. Compared with the 3D target, the 2D planar calibration system has plenty of advantages. The 2D target calibration system is easy to be manufactured and suitable for the on-site calibration. In addition, the 2D planar calibration system provides sufficient calibration information in a convenient way. The 1D target is also easy to be made than the 2D target and 3D target, however, the measurement accuracy of 1D target is lower than the 2D calibration system and the 3D calibration system because of the lack of information. Thus, the 2D planar calibration system is adopted in this study.

A reconstruction method of the 3D laser projective point is proposed for the measurement adopting the line-structured-light. The 3D reconstruction method of the laser projective point is cataloged into four parts. Firstly, the laser line of the projector lies on the plane that is coincident with the 2D calibration target plane. Therefore, on the same plane, non-collinear points can be extracted to construct a projection invariant. Secondly, a laser projective point and five non-collinear points on the target are randomly extracted from the captured image. Similarly, the invariant with the same points is calculated in the camera coordinate system. For the same points, the invariant in the image coordinate system is equal to the one in the camera coordinate system. According to the idea above, homogeneous linear equations are constructed and the closed form solution is determined by the decompositions of singular values. Thirdly, the optimization function is designed to promote the reconstruction accuracy by minimizing the parameterized re-projection errors of the laser point and the target points. The 3D laser points are reconstructed in the world coordinate system. Finally, the effects of the image quantity, the lens distortion and noises are experimentally evaluated by comparing the differences between the reconstruction displacements of the laser points and the real displacements.

The following paper is outlined as follows: Section 2, Methods, present the reconstruction approach consisting of the closed form solution and optimization solution. Section 3, Results, provide the experimentation to verify the reconstruction method. The factors, lens distortion, noises and application cases are considered in the section. Section 4, Discussion, evaluates the reconstruction method according to the absolute and relative errors. Section 5, Summary, concludes the paper.

## Methods

The reconstruction method of the projective point of the laser line includes two main components. The first component is to calibrate the camera, which has been accurately solved by Zhang^21^. The intrinsic and extrinsic parameters of a camera, including rotation matrix and translation vector, are provided by the calibration results. We consequently focus on the second component, to construct the projection invariant according to the laser projective point and five target points. The closed form solution of the laser projective point is achieved in this section and further improved by the optimization function.

**Figure 1:**
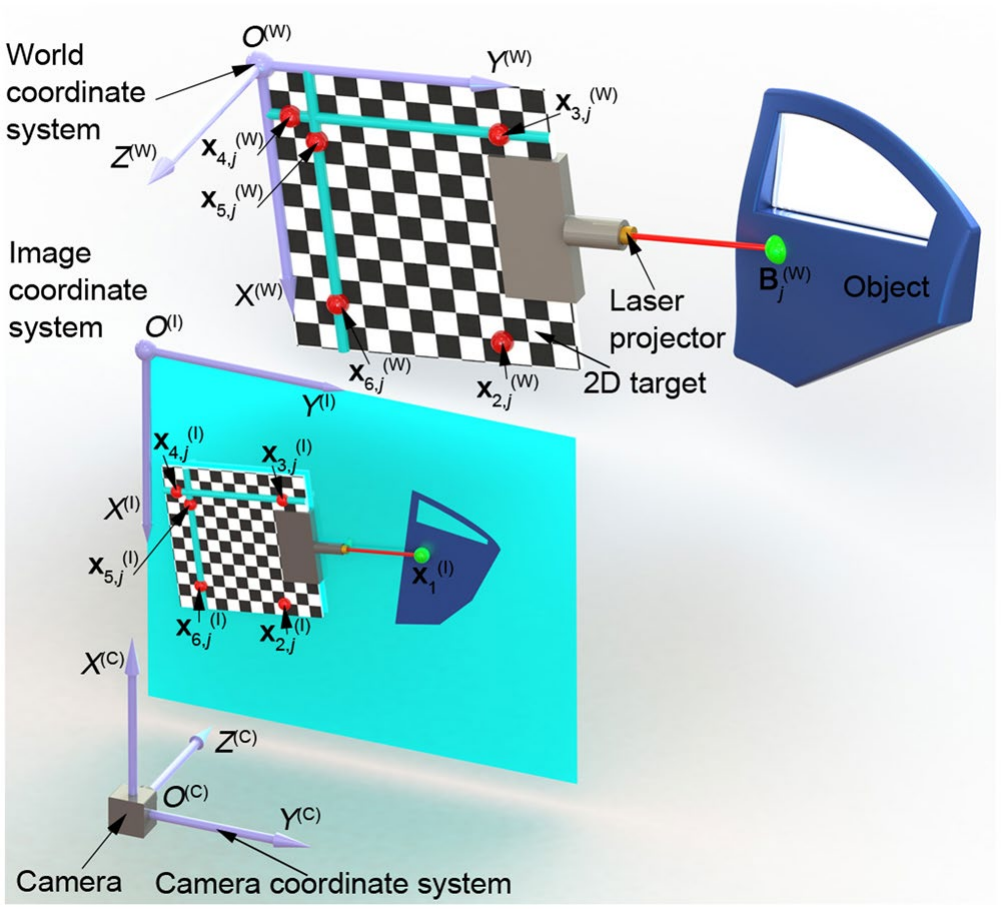
The reconstruction method of the laser projective point with the projection invariant generated from the five points on a 2D target.

**Figure 2:**
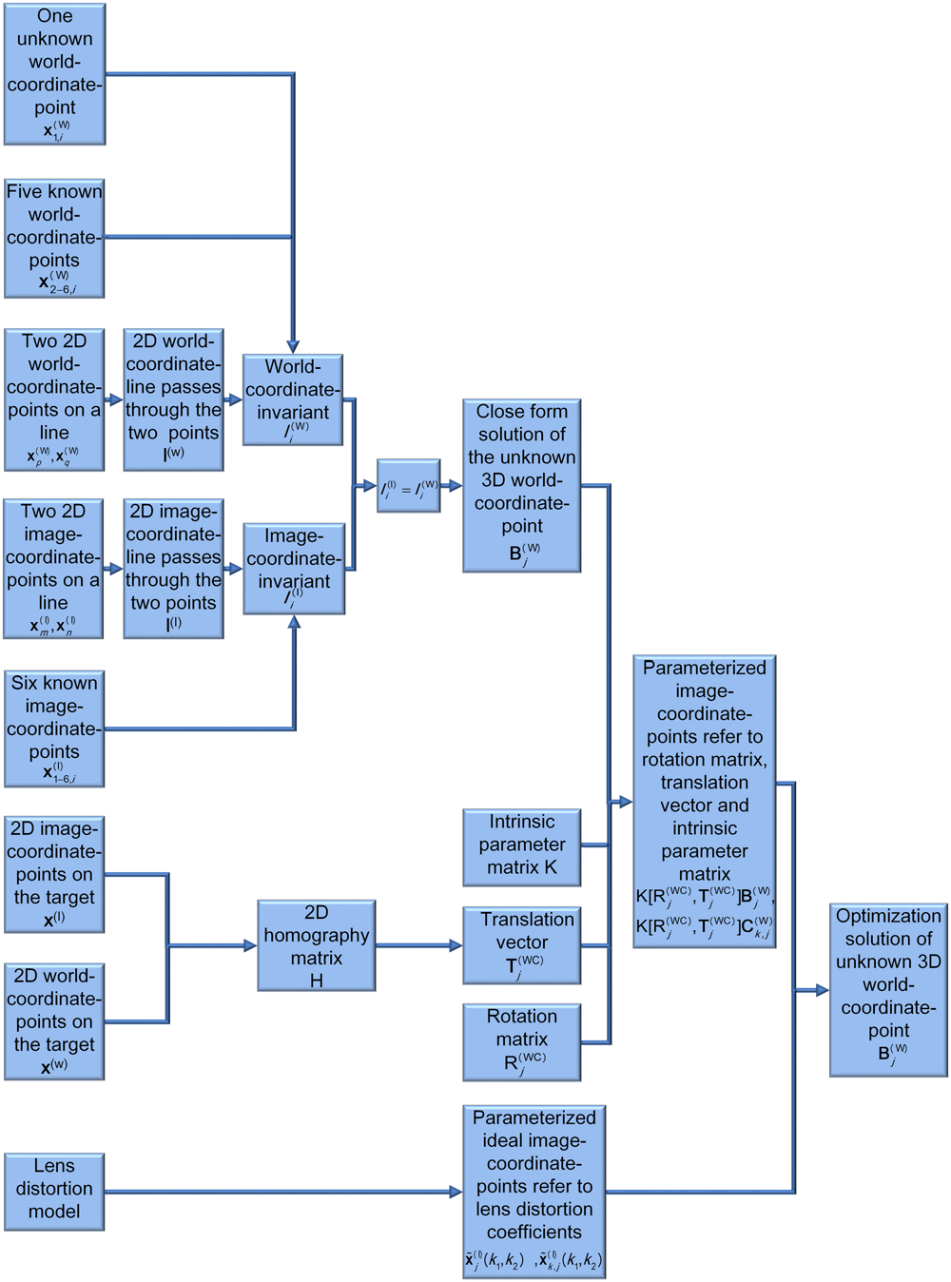
The block diagram of the reconstruction approach of the laser projective point.

The reconstruction model of the laser projective point on an object is illustrated in Fig. 1. The reconstruction approach is interpreted by the block diagram in Fig. 2. The camera, the target and the image plane define the three coordinate systems *O* ^(C)^-*X* ^(C)^ *Y* ^(C)^ *Z* ^(C)^, *O* ^(W)^-*X* ^(W)^ *Y* ^(W)^ *Z* ^(W)^ and *O* ^(I)^-*X* ^(I)^ *Y* ^(I)^, respectively. In the camera-laser-line-based measurement system, a 2D target is employed to perform the two aims, the camera calibration and the projective point reconstruction of a laser line. A laser projector is connected to the target. The laser line of the projector lies on the *O* ^(W)^-*X* ^(W)^ *Y* ^(W)^ plane that is the identical one of the target. Considering the coplanarity of the laser projective point, the laser line, the target plane and the target feature points, there is a projective invariant derived from the laser projective point on the measured object and the five points on the target. The laser projective point and the five points on the target should be non-collinear to avoid the linear dependence. The projective invariant in the world coordinate system is identical to the projective invariant of the laser projective point and the five mapping target points in the image coordinate system. The laser projective points and the mapping target feature points are extracted by the Harris method^25^.

The laser line projector is fixed on a planar target attached with the world coordinate system. The laser line is located on the *O* ^(W)^-*X* ^(W)^ *Y* ^(W)^ plane of the world coordinate system. Thus, the projective point of the laser line is also placed in the *O* ^(W)^-*X* ^(W)^ *Y* ^(W)^ plane. In the projection geometry, the projective point **x** ^(I)^ in the image coordinate system and the projective line **l** ^(I)^ in the image coordinate system can be given by ref. 26

1 $${{\bf{x}}}^{({\rm{I}})}={\rm{H}}\,{{\bf{x}}}^{({\rm{W}})}$$

2 $${{\bf{l}}}^{({\rm{I}})}={{\rm{H}}}^{-{\rm{T}}}\,{{\bf{l}}}^{({\rm{W}})}$$

where **x** ^(W)^ is the 2D homogenous coordinates of a point on the *O* ^(W)^-*X* ^(W)^ *Y* ^(W)^ plane, **l** ^(W)^ is the 2D homogenous coordinates of a line on the *O* ^(W)^-*X* ^(W)^ *Y* ^(W)^ plane, H is the homography matrix from the world coordinate system to the image coordinate system, the superscript (I) indicates the points or lines defined in the image coordinate system, the superscript (W) indicates the points or lines defined in the world coordinate system.

The 2D line on the *O* ^(W)^-*X* ^(W)^ *Y* ^(W)^ plane, **l** ^(W)^, can be denoted by ref. 26

3 $${{\bf{l}}}^{({\rm{W}})}={{\bf{x}}}_{p}^{({\rm{W}})}\times {{\bf{x}}}_{q}^{({\rm{W}})}$$

where ${{\bf{x}}}_{p}^{({\rm{W}})}$ and ${{\bf{x}}}_{q}^{({\rm{W}})}$ are two 2D points on the line **l** ^(W)^.

The projective line **l** ^(I)^ in the image coordinate system is given by ref. 26

4 $${{\bf{l}}}^{({\rm{I}})}={{\bf{x}}}_{m}^{({\rm{I}})}\times {{\bf{x}}}_{n}^{({\rm{I}})}$$

where ${{\bf{x}}}_{m}^{({\rm{I}})}$ and ${{\bf{x}}}_{n}^{({\rm{I}})}$ are two 2D points on the line **l** ^(I)^.

A projection variable ${I}_{i}^{({\rm{I}})}$ generated from five 2D points on the 2D target and a laser projective point is constructed by

5 $$\begin{array}{rcl}{I}_{i}^{({\rm{I}})} & = & [{({{\bf{x}}}_{3,i}^{({\rm{I}})}\times {{\bf{x}}}_{4,i}^{({\rm{I}})})}^{{\rm{T}}}\,{{\bf{x}}}_{1}^{({\rm{I}})}]\,[{({{\bf{x}}}_{5,i}^{({\rm{I}})}\times {{\bf{x}}}_{6,i}^{({\rm{I}})})}^{{\rm{T}}}\,{{\bf{x}}}_{2,i}^{({\rm{I}})}]\\  &  & {[{({{\bf{x}}}_{3,i}^{({\rm{I}})}\times {{\bf{x}}}_{4,i}^{({\rm{I}})})}^{{\rm{T}}}{{\bf{x}}}_{2,i}^{({\rm{I}})}]}^{-1}\,{[{({{\bf{x}}}_{5,i}^{({\rm{I}})}\times {{\bf{x}}}_{6,i}^{({\rm{I}})})}^{{\rm{T}}}{{\bf{x}}}_{1}^{({\rm{I}})}]}^{-1}\end{array}$$

where ${{\bf{x}}}_{1}^{({\rm{I}})}$ is the projective point of the laser line, ${{\bf{x}}}_{2-6,i}^{({\rm{I}})}$ are the five point coordinates on the 2D target in the image coordinate system, *i* is the group number of the five points on the target in an captured image.

Stacking Eqs (1)–(5), we have the projection invariant

6 $$\begin{array}{rcl}{I}_{i}^{({\rm{I}})}={I}_{i}^{({\rm{W}})} & = & [{({{\bf{x}}}_{3,i}^{({\rm{W}})}\times {{\bf{x}}}_{4,i}^{({\rm{W}})})}^{{\rm{T}}}\,{{\bf{x}}}_{1}^{({\rm{W}})}]\,[{({{\bf{x}}}_{5,i}^{({\rm{W}})}\times {{\bf{x}}}_{6,i}^{({\rm{W}})})}^{{\rm{T}}}\,{{\bf{x}}}_{2,i}^{({\rm{W}})}]\\  &  & {[{({{\bf{x}}}_{3,i}^{({\rm{W}})}\times {{\bf{x}}}_{4,i}^{({\rm{W}})})}^{{\rm{T}}}{{\bf{x}}}_{2,i}^{({\rm{W}})}]}^{-1}\,{[{({{\bf{x}}}_{5,i}^{({\rm{W}})}\times {{\bf{x}}}_{6,i}^{({\rm{W}})})}^{{\rm{T}}}{{\bf{x}}}_{1}^{({\rm{W}})}]}^{-1}\end{array}$$

where ${{\bf{x}}}_{1}^{({\rm{W}})}$ is the unknown projective point of the laser line, ${{\bf{x}}}_{2-6,i}^{({\rm{W}})}$ are the five point coordinates on the 2D target in the world coordinate system, *i* is the group number of the five points on the target.

The projection invariant ${I}_{i}^{({\rm{I}})}$ in the image coordinate system is identical to the one in the world coordinate system, ${I}_{i}^{({\rm{W}})}$, then

7 $$\begin{array}{l}\{[{({{\bf{x}}}_{3,i}^{({\rm{I}})}\times {{\bf{x}}}_{4,i}^{({\rm{I}})})}^{{\rm{T}}}\,{{\bf{x}}}_{1}^{({\rm{I}})}]\,[{({{\bf{x}}}_{5,i}^{({\rm{I}})}\times {{\bf{x}}}_{6,i}^{({\rm{I}})})}^{{\rm{T}}}\,{{\bf{x}}}_{2,i}^{({\rm{I}})}]\,[{({{\bf{x}}}_{3,i}^{({\rm{W}})}\times {{\bf{x}}}_{4,i}^{({\rm{W}})})}^{{\rm{T}}}\,{{\bf{x}}}_{2,i}^{({\rm{W}})}]\,{({{\bf{x}}}_{5,i}^{({\rm{W}})}\times {{\bf{x}}}_{6,i}^{({\rm{W}})})}^{{\rm{T}}}\\ \quad -[{({{\bf{x}}}_{3,i}^{({\rm{I}})}\times {{\bf{x}}}_{4,i}^{({\rm{I}})})}^{{\rm{T}}}\,{{\bf{x}}}_{2,i}^{({\rm{I}})}]\,[{({{\bf{x}}}_{5,i}^{({\rm{I}})}\times {{\bf{x}}}_{6,i}^{({\rm{I}})})}^{{\rm{T}}}\,{{\bf{x}}}_{1}^{({\rm{I}})}]\,[{({{\bf{x}}}_{5,i}^{({\rm{W}})}\times {{\bf{x}}}_{6,i}^{({\rm{W}})})}^{{\rm{T}}}\,{{\bf{x}}}_{2,i}^{({\rm{W}})}]\,{({{\bf{x}}}_{3,i}^{({\rm{W}})}\times {{\bf{x}}}_{4,i}^{({\rm{W}})})}^{{\rm{T}}}\}\,{{\bf{x}}}_{1}^{({\rm{W}})}=0\end{array}$$

For different point combinations in the image, Eq. (7) is represented by a matrix form as

8 $${\rm{V}}{{\bf{x}}}_{1}^{({\rm{W}})}={\bf{0}}$$

where **0**is the zero vector,

$$\begin{array}{ccc}{\rm{V}} & = & [\begin{array}{c}[{({{\bf{x}}}_{3,1}^{({\rm{I}})}\times {{\bf{x}}}_{4,1}^{({\rm{I}})})}^{{\rm{T}}}{{\bf{x}}}_{1}^{({\rm{I}})}][{({{\bf{x}}}_{5,1}^{({\rm{I}})}\times {{\bf{x}}}_{6,1}^{({\rm{I}})})}^{{\rm{T}}}{{\bf{x}}}_{2,1}^{({\rm{I}})}][{({{\bf{x}}}_{3,1}^{({\rm{W}})}\times {{\bf{x}}}_{4,1}^{({\rm{W}})})}^{{\rm{T}}}{{\bf{x}}}_{2,1}^{({\rm{W}})}]{({{\bf{x}}}_{5,1}^{({\rm{W}})}\times {{\bf{x}}}_{6,1}^{({\rm{W}})})}^{{\rm{T}}}\\ \left[{({{\bf{x}}}_{3,2}^{({\rm{I}})}\times {{\bf{x}}}_{4,2}^{({\rm{I}})})}^{{\rm{T}}}{{\bf{x}}}_{1}^{({\rm{I}})}\right][{({{\bf{x}}}_{5,2}^{({\rm{I}})}\times {{\bf{x}}}_{6,2}^{({\rm{I}})})}^{{\rm{T}}}{{\bf{x}}}_{2,1}^{({\rm{I}})}][{({{\bf{x}}}_{3,2}^{({\rm{W}})}\times {{\bf{x}}}_{4,2}^{({\rm{W}})})}^{{\rm{T}}}{{\bf{x}}}_{2,2}^{({\rm{W}})}]{({{\bf{x}}}_{5,2}^{({\rm{W}})}\times {{\bf{x}}}_{6,2}^{({\rm{W}})})}^{{\rm{T}}}\\ ?\\ \left[{({{\bf{x}}}_{3,i}^{({\rm{I}})}\times {{\bf{x}}}_{4,i}^{({\rm{I}})})}^{{\rm{T}}}{{\bf{x}}}_{1}^{({\rm{I}})}\right][{({{\bf{x}}}_{5,{\rm{i}}}^{({\rm{I}})}\times {{\bf{x}}}_{6,{\rm{i}}}^{({\rm{I}})})}^{{\rm{T}}}{{\bf{x}}}_{2,{i}}^{({\rm{I}})}][{({{\bf{x}}}_{3,{i}}^{({\rm{W}})}\times {{\bf{x}}}_{4,{i}}^{({\rm{W}})})}^{{\rm{T}}}{{\bf{x}}}_{2,{i}}^{({\rm{W}})}]{({{\bf{x}}}_{5,{i}}^{({\rm{W}})}\times {{\bf{x}}}_{6,{i}}^{({\rm{W}})})}^{{\rm{T}}}\end{array}\\  &  & \begin{array}{c}-[{({{\bf{x}}}_{3,1}^{({\rm{I}})}\times {{\bf{x}}}_{4,1}^{({\rm{I}})})}^{{\rm{T}}}{{\bf{x}}}_{2,1}^{({\rm{I}})}][{({{\bf{x}}}_{5,1}^{({\rm{I}})}\times {{\bf{x}}}_{6,1}^{({\rm{I}})})}^{{\rm{T}}}{{\bf{x}}}_{1}^{({\rm{I}})}][{({{\bf{x}}}_{5,1}^{({\rm{W}})}\times {{\bf{x}}}_{6,1}^{({\rm{W}})})}^{{\rm{T}}}{{\bf{x}}}_{2,1}^{({\rm{W}})}]{({{\bf{x}}}_{3,1}^{({\rm{W}})}\times {{\bf{x}}}_{4,1}^{({\rm{W}})})}^{{\rm{T}}}\\ -[{({{\bf{x}}}_{3,2}^{({\rm{I}})}\times {{\bf{x}}}_{4,2}^{({\rm{I}})})}^{{\rm{T}}}{{\bf{x}}}_{2,2}^{({\rm{I}})}][{({{\bf{x}}}_{5,2}^{({\rm{I}})}\times {{\bf{x}}}_{6,2}^{({\rm{I}})})}^{{\rm{T}}}{{\bf{x}}}_{1}^{({\rm{I}})}][{({{\bf{x}}}_{5,2}^{({\rm{W}})}\times {{\bf{x}}}_{6,2}^{({\rm{W}})})}^{{\rm{T}}}{{\bf{x}}}_{2,2}^{({\rm{W}})}]{({{\bf{x}}}_{3,2}^{({\rm{W}})}\times {{\bf{x}}}_{4,2}^{({\rm{W}})})}^{{\rm{T}}}\\ ?\\ -[{({{\bf{x}}}_{3,i}^{({\rm{I}})}\times {{\bf{x}}}_{4,i}^{({\rm{I}})})}^{{\rm{T}}}{{\bf{x}}}_{2,{i}}^{({\rm{I}})}][{({{\bf{x}}}_{5,{i}}^{({\rm{I}})}\times {{\bf{x}}}_{6,{i}}^{({\rm{I}})})}^{{\rm{T}}}{{\bf{x}}}_{1}^{({\rm{I}})}][{({{\bf{x}}}_{5,{i}}^{({\rm{W}})}\times {{\bf{x}}}_{6,{i}}^{({\rm{W}})})}^{{\rm{T}}}{{\bf{x}}}_{2,{i}}^{({\rm{W}})}]{({{\bf{x}}}_{3,{i}}^{({\rm{W}})}\times {{\bf{x}}}_{4,{i}}^{({\rm{W}})})}^{{\rm{T}}}\end{array}]\end{array}$$

The unknown projective point ${{\bf{x}}}_{1}^{({\rm{W}})}$ in Eq. (8) is solved by the singular value decomposition^27^. As ${{\bf{x}}}_{1}^{({\rm{W}})}$ is located on the *O* ^(W)^-*X* ^(W)^ *Y* ^(W)^ plane, in the *j*th image, the 3D projective point of the laser line is ${{\bf{B}}}_{j}^{({\rm{W}})}={[{{\bf{x}}}_{1}^{({\rm{W}})}(1),{{\bf{x}}}_{1}^{({\rm{W}})}(2),0,1]}^{{\rm{T}}}$ in the world coordinate system.

In order to transform the 3D projective point from the free world coordinate system to the stationary camera coordinate system, the 3D projective point ${{\bf{B}}}_{j}^{({\rm{W}})}$ is left multiplied by the calibrated rotation matrix ${{\rm{R}}}_{j}^{({\rm{WC}})}$ and the translation vector ${{\bf{T}}}_{j}^{({\rm{WC}})}$ as ref. 26

9 $${{\bf{B}}}_{j}^{({\rm{C}})}=[{{\rm{R}}}_{{j}}^{({\rm{W}}{\rm{C}})},{{\bf{T}}}_{{j}}^{({\rm{W}}{\rm{C}})}]{{\bf{B}}}_{{j}}^{({\rm{W}})}$$

where ${{\bf{B}}}_{j}^{({\rm{C}})}$ is the 3D projective point of the laser line in the camera coordinate system.

Considering the lens distortion coefficients *k*_1_ and *k*_2_, the ideal image points and the distortion image points are expressed by ref. 21

10 $$\begin{array}{rcl}u({k}_{1},{k}_{2}) & = & \frac{\hat{u}+{u}_{0}[{k}_{1}({x}^{2}+{y}^{2})+{k}_{2}{({x}^{2}+{y}^{2})}^{2}]}{1+{k}_{1}({x}^{2}+{y}^{2})+{k}_{2}{({x}^{2}+{y}^{2})}^{2}}\\ v({k}_{1},{k}_{2}) & = & \frac{\hat{v}+{v}_{0}[{k}_{1}({x}^{2}+{y}^{2})+{k}_{2}{({x}^{2}+{y}^{2})}^{2}]}{1+{k}_{1}({x}^{2}+{y}^{2})+{k}_{2}{({x}^{2}+{y}^{2})}^{2}}\end{array}$$

where *u*, *v* are the ideal pixel coordinates, *u*_0_, *v*_0_ are the coordinates of the principal point, *x*, *y* are the ideal image coordinates in the camera coordinate system, $\hat{u}$, $\hat{v}$ are the real pixel coordinates. The initial solutions of the distortion coefficients *k*_1_ and *k*_2_ are explained in ref. 21.

Equation (9) provides the initial solution of the 3D projective point ${{\bf{B}}}_{j}^{({\rm{C}})}$ of the laser line. It can be improved by the optimization process. The 3D laser projective point ${{\bf{B}}}_{j}^{({\rm{W}})}$ and the target feature points ${{\bf{C}}}_{k,j}^{({\rm{W}})}$ are re-projected to the image coordinate system. The ideal laser projective point ${\tilde{{\bf{x}}}}_{j}^{({\rm{I}})}({k}_{1},{k}_{2})$ and the ideal target feature points ${\tilde{{\bf{x}}}}_{k,j}^{({\rm{I}})}({k}_{1},{k}_{2})$ are parameterized by Eq. (10). The re-projected points should approach the ideal points in the image. Therefore, the differences of the re-projected points and the ideal points in the image coordinate system are contributed by minimizing the optimization function

11 $$({{\bf{B}}}_{j}^{({\rm{W}})},{\rm{K}},{{\rm{R}}}_{j}^{({\rm{WC}})},{{\bf{T}}}_{j}^{({\rm{WC}})},{k}_{1},{k}_{2})={\rm{\arg }}\,\{{\rm{\min }}\,[\sum _{j=1}^{N}\sum _{k=1}^{M}({\Vert {\rm{K}}[{{\rm{R}}}_{j}^{({\rm{WC}})},{{\bf{T}}}_{j}^{({\rm{WC}})}]{{\bf{B}}}_{j}^{({\rm{W}})}-{\tilde{{\bf{x}}}}_{j}^{({\rm{I}})}({k}_{1},{k}_{2})\Vert }^{2}+{\Vert {\rm{K}}[{{\rm{R}}}_{j}^{({\rm{WC}})},{{\bf{T}}}_{j}^{({\rm{WC}})}]{{\bf{C}}}_{k,j}^{({\rm{W}})}-{\tilde{{\bf{x}}}}_{k,j}^{({\rm{I}})}({k}_{1},{k}_{2})\Vert }^{2})]\}$$

where ${\tilde{{\bf{x}}}}_{j}^{({\rm{I}})}({k}_{1},{k}_{2})$ is the ideal laser projective point parameterized by *k*_1_ and *k*_2_ in the *j*th image, ${\tilde{{\bf{x}}}}_{k,j}^{({\rm{I}})}({k}_{1},{k}_{2})$ is the *k*th ideal target feature points parameterized by *k*_1_ and *k*_2_ in the *j*th image, K is the intrinsic parameter matrix of the calibrated camera, *N* is the number of images, *M* is the number of the target feature points ${{\bf{C}}}_{k,j}^{({\rm{W}})}$ in the *j*th image.

### Data availability

The datasets generated during the current study are available in the Figshare repository, https://doi.org/10.6084/m9.figshare.5074069.v1.

## Results

**Figure 3:**
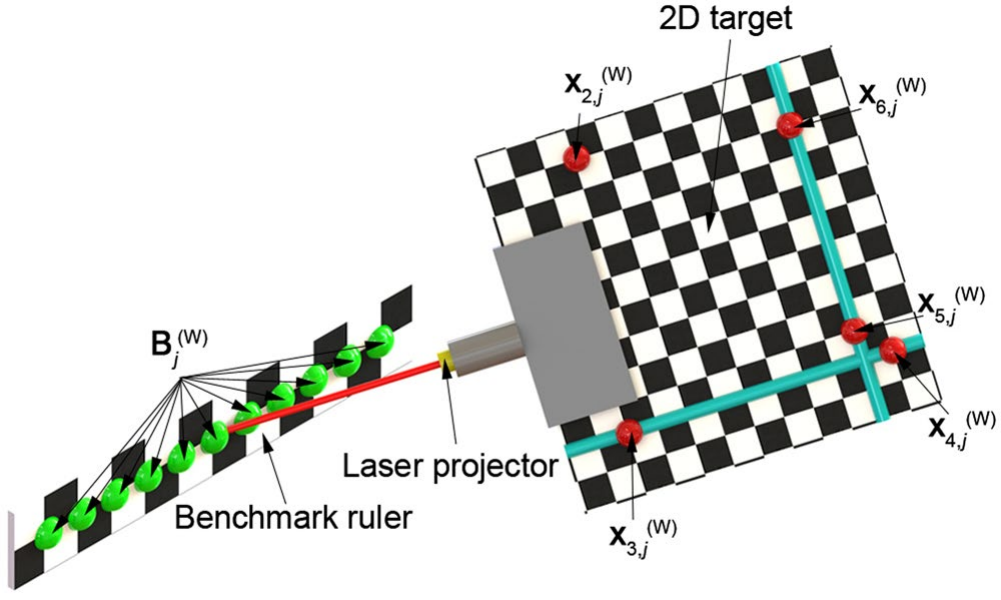
The verification method of the reconstruction results using a benchmark ruler with the checkerboard pattern.

The 3D reconstruction precision of the projective point of the laser line cannot be directly evaluated as there is no benchmark of the point in the camera coordinate system. However, the displacement between two projective points in the world coordinate system is identical to the one in the camera coordinate system. Thus, the displacement of two projective points is considered as the relative benchmark for the reconstruction. The verification principle of the method is illustrated in Fig. 3. The ruler with the checkerboard pattern is chosen as the benchmark of the test. The feature point coordinates on the benchmark ruler can be reconstructed by the laser projector system.

The projective laser point ${{\bf{B}}}_{j}^{({\rm{W}})}$ in the world coordinate system is achieved by the optimization process. It is transformed to the projective laser point ${{\bf{B}}}_{j}^{({\rm{C}})}$ in the camera coordinate system. The reconstruction displacement error of two projective laser points is

12 $${E}_{j}^{({\rm{C}})}=\Vert {{\bf{B}}}_{j}^{({\rm{C}})}-{{\bf{B}}}_{j-1}^{({\rm{C}})}\Vert -{B}_{0}$$

where ${E}_{j}^{({\rm{C}})}$ is the reconstruction displacement error, *B*_0_ is the benchmark of the point displacement.

**Figure 4:**
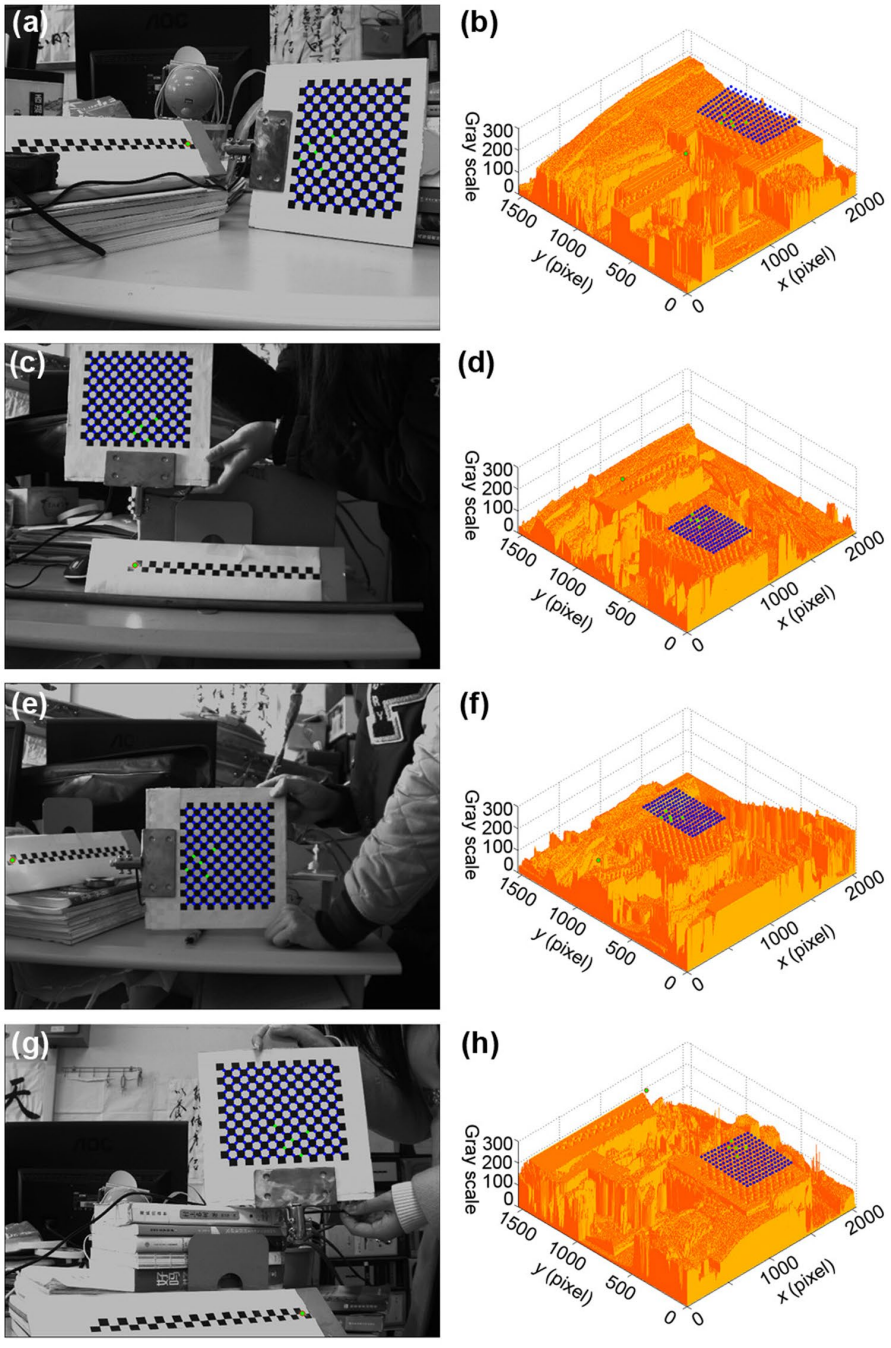
The Harris recognition results of the laser points and 2D target points in the four experiments. The laser point and five target points are marked by green. (**a**,**c**,**e**,**g**) The recognition results of the laser points and the 2D target points in the experiments. (**b**,**d**,**f**,**h**) The laser points and 2D target points in the maps of gray-scale distributions of (**a**,**c**,**e**,**g**).

In the experiments, a 140 mm × 160 mm target is designed with 13 × 15 points. The distance of adjacent corners is 10 mm. 2048 × 1536 resolution is used in the experiments. Four groups of experiments are performed to reconstruct the displacements among the 3D laser points and verify the accuracy of the reconstruction. The Harris corner recognition results and the gray-scale distributions in four groups of experiments are shown in Fig. 4. The blue points indicate the recognition results of the 2D target points. In addition, there are six green points in every image. Five of them are non-collinear points that are randomly selected in the 2D target. The other one is the laser projective point.

**Figure 5:**
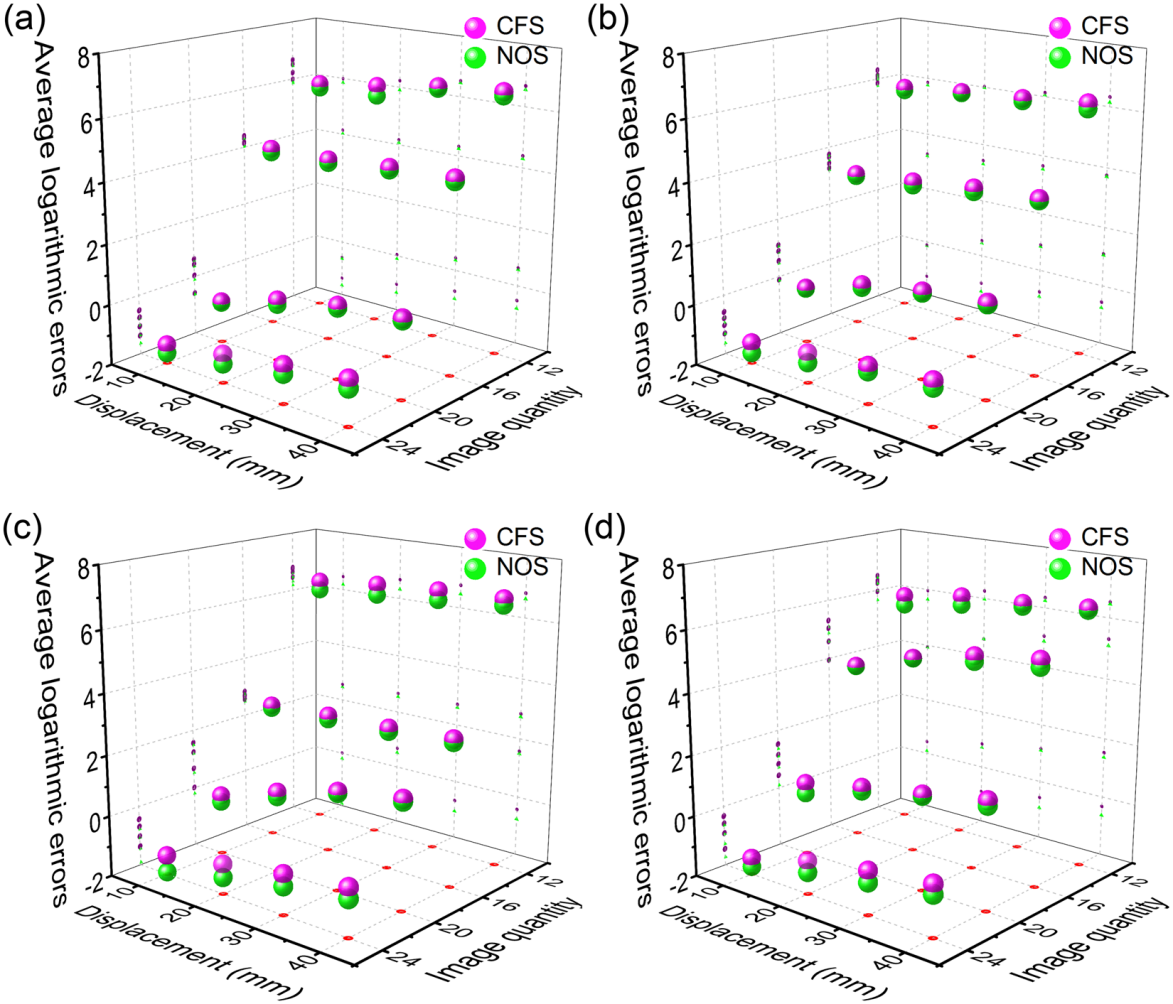
The average logarithmic errors of the close form solution (CFS) and the nonlinear optimization solution (NOS) without considering the lens distortion. The numbers of images are 12, 16, 20 and 24, respectively. The displacements are 10 mm, 20 mm, 30 mm and 40 mm, respectively. The purple balls represent the CFS test errors. The green balls show the NOS error results.

Figure 5 shows the reconstruction errors of laser points of the nonlinear optimization solution (NOS) and the close form solution (CFS), without considering the distortion in the four groups of experiments. Figure 5(a–d) describe the reconstruction errors of the four groups, respectively. In the first group, when the displacement is 10 mm and the image quantities increase from 12 to 24, the average logarithmic errors of CFS are 5.99 lnmm, 4.15 lnmm, −0.58 lnmm and −1.37 lnmm, respectively. The average logarithmic errors of NOS are 5.84 lnmm, 4.01 lnmm, −0.64 lnmm and −1.66 lnmm, respectively. The average logarithmic errors of CFS are respectively 6.23 lnmm, 4.17 lnmm, 0.04 lnmm and −1.06 lnmm while the displacement is 20 mm and the image quantities increase. The corresponding average logarithmic errors of NOS are 5.89 lnmm, 4.06 lnmm, −0.12 lnmm and −1.37 lnmm, respectively. When the displacement is 30 mm and the image quantities increase, the average logarithmic errors of CFS are 6.52 lnmm, 4.32 lnmm, 0.44 lnmm and −0.75 lnmm, respectively. The related average logarithmic errors of NOS are 6.45 lnmm, 4.24 lnmm, 0.35 lnmm and −1.03 lnmm, respectively. The average logarithmic errors of CFS are 6.67 lnmm, 4.39 lnmm, 0.62 lnmm and −0.51 lnmm while the displacement is 40 mm and the image quantities increase. The corresponding average logarithmic errors of NOS are 6.54 lnmm, 4.29 lnmm, 0.55 lnmm and −0.80 lnmm.

In the second group, when the displacement of 10 mm and the image quantities rise from 12 to 24, the means of logarithmic errors of CFS are 5.86 lnmm, 3.27 lnmm, −0.11 lnmm and −1.30 lnmm, respectively. The corresponding means of the logarithmic errors of NOS are 5.75 lnmm, 3.19 lnmm, −0.15 lnmm and −1.65 lnmm, respectively. When the displacement is 20 mm and the image quantities grow up, the average logarithmic errors of CFS are 6.02 lnmm, 3.44 lnmm, 0.56 lnmm and −1.02 lnmm, respectively. The average logarithmic errors of NOS are 5.98 lnmm, 3.31 lnmm, 0.45 lnmm and −1.33 lnmm, respectively. When the displacement is 30 mm and the image quantities grow up, the means of logarithmic errors of CFS are 6.13 lnmm, 3.66 lnmm, 0.90 lnmm and −0.78 lnmm, respectively. The means of logarithmic errors of NOS are 6.05 lnmm, 3.54 lnmm, 0.84 lnmm and −0.95 lnmm, respectively. The average logarithmic errors of CFS are 6.32 lnmm, 3.75 lnmm, 1.10 lnmm and −0.57 lnmm while the displacement is 40 mm and the image quantities grow up. The means of logarithmic errors of NOS are 6.13 lnmm, 3.70 lnmm, 1.05 lnmm and −0.77 lnmm, respectively.

In the third group, when the displacement is 10 mm and the image quantities increase from 12 to 24, the means of the logarithmic errors of CFS are 6.46 lnmm, 2.57 lnmm, 0.01 lnmm and −1.36 lnmm, respectively. The average logarithmic errors of NOS are 6.17 lnmm, 2.47 lnmm, −0.2 lnmm and −1.93 lnmm, respectively. The means of the logarithmic errors of CFS are 6.64 lnmm, 2.67 lnmm, 0.68 lnmm and −1.01 lnmm while the displacement is 20 mm and the image quantities increase. The average logarithmic errors of NOS are 6.31 lnmm, 2.54 lnmm, 0.52 lnmm and −1.45 lnmm, respectively. When the displacement is 30 mm and the image quantities increase, the means of logarithmic errors of CFS are 6.75 lnmm, 2.72 lnmm, 1.25 lnmm and −0.68 lnmm, respectively. The average logarithmic errors of NOS are 6.45 lnmm, 2.60 lnmm, 1.19 lnmm and −1.07 lnmm, respectively. When the displacement is 40 mm and the image quantities increase, the means of logarithmic errors of CFS are 6.80 lnmm, 2.84 lnmm, 1.57 lnmm and −0.43 lnmm, respectively. The average logarithmic errors of NOS are 6.60 lnmm, 2.73 lnmm, 1.48 lnmm and −0.78 lnmm, respectively.

In the fourth group, when the displacement is 10 mm and the image quantities rise from 12 to 24, the average logarithmic errors of CFS are 5.96 lnmm, 3.95 lnmm, 0.44 lnmm and −1.45 lnmm. The corresponding means of the average logarithmic errors of NOS are 5.64 lnmm, 3.92 lnmm, 0.08 lnmm and −1.74 lnmm, respectively. The average logarithmic errors of CFS are 6.26 lnmm, 4.59 lnmm, 0.84 lnmm and −0.91 lnmm, with the displacement of 20 mm and the image quantities grow up. The means of the average logarithmic errors of NOS are 5.96 lnmm, 4.58 lnmm, 0.70 lnmm and −1.29 lnmm, respectively. When the displacement is 30 mm and the image quantities grow up, the average logarithmic errors of CFS are 6.34 lnmm, 5.05 lnmm, 1.16 lnmm and −0.57 lnmm, respectively. The corresponding means of the average logarithmic errors of NOS are 6.23 lnmm, 4.87 lnmm, 1.10 lnmm and −0.95 lnmm. The average logarithmic errors of CFS are 6.51 lnmm, 5.31 lnmm, 1.53 lnmm and −0.33 lnmm with the displacement of 40 mm and the image quantities grow up. The corresponding average logarithmic errors of NOS are 6.44 lnmm, 5.09 lnmm, 1.36 lnmm and −0.64 lnmm, respectively.

It is apparent from the experimental results in Fig. 5 that the error gradually increases with the increasing displacement with the same image quantity. The displacement of the proposed optimization method with lens distortion is closer to the real displacement than the optimization method without considering the distortion. When the image number is 24, the reconstruction error of the displacement approaches its minimum. As the image number increases, the camera calibration is more accurate. Accordingly, the difference between the reconstructed displacement of the laser points and the actual displacement declines with the increasing image number. Therefore, in the following experiments, 24 images are adopted to calibrate the camera and analyze the test errors.

**Figure 6:**
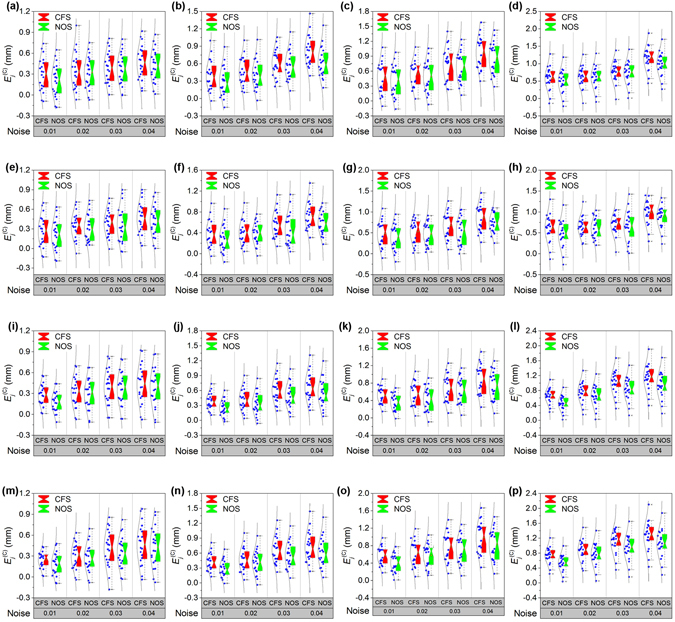
Experimental results of the close form solution (CFS) and the nonlinear optimization solution (NOS) affected by the noises of 0.01, 0.02, 0.03 and 0.04, respectively. (**a**–**d**) The first group of comparison results of CFS and NOS in the displacements of 10 mm, 20 mm, 30 mm and 40 mm, respectively. (**e**–**h**) The second group of comparison results of CFS and NOS in the displacements of 10 mm, 20 mm, 30 mm and 40 mm, respectively. (**i**–**l**) The third group of comparison results of CFS and NOS in the displacements of 10 mm, 20 mm, 30 mm and 40 mm, respectively. (**m**–**p**) The fourth group of comparison results of CFS and NOS in the displacements of 10 mm, 20 mm, 30 mm and 40 mm, respectively.

In the reconstruction of the displacement between the projection laser points, noise is an indispensable factor to be evaluated. The noise effects on the displacement reconstructions are investigated in the experiments. Four kinds of Gaussian noises are added to test the influences on the reconstruction errors. The experimental results of NOS and CFS without considering the lens distortion in the four groups are illustrated in Fig. 6. Four groups of experiments are performed to compare the effects of the noises and displacements. The statistical data are shown in Table 1. It is evident that the reconstruction error shows a stable growing trend with the increasing noise in the above experimental results. The errors of NOS are smaller than the errors of CFS under the same noise level. Therefore, NOS provides a higher accuracy. Under the same noise and the same optimization condition, the error values increase with the increasing displacement.

**Table 1:** The error means of the close form solution (CFS) and the nonlinear optimization solution (NOS) affected by the noises related to Fig. 6.

Group	Displacement	Solution method	Error mean			
			Noise 0.01	Noise 0.02	Noise 0.03	Noise 0.04
1	10 mm	Close form solution	0.30	0.35	0.40	0.47
		Nonlinear optimization solution	0.21	0.32	0.38	0.42
	20 mm	Close form solution	0.39	0.46	0.61	0.82
		Nonlinear optimization solution	0.28	0.40	0.56	0.61
	30 mm	Close form solution	0.46	0.54	0.75	0.97
		Nonlinear optimization solution	0.40	0.49	0.68	0.85
	40 mm	Close form solution	0.58	0.63	0.78	1.16
		Nonlinear optimization solution	0.50	0.61	0.74	0.99
2	10 mm	Close form solution	0.27	0.35	0.39	0.46
		Nonlinear optimization solution	0.20	0.31	0.36	0.42
	20 mm	Close form solution	0.38	0.41	0.56	0.73
		Nonlinear optimization solution	0.27	0.39	0.45	0.59
	30 mm	Close form solution	0.48	0.53	0.67	0.86
		Nonlinear optimization solution	0.39	0.47	0.55	0.74
	40 mm	Close form solution	0.61	0.65	0.71	0.99
		Nonlinear optimization solution	0.48	0.61	0.66	0.89
3	10 mm	Close form solution	0.27	0.32	0.39	0.43
		Nonlinear optimization solution	0.18	0.29	0.37	0.39
	20 mm	Close form solution	0.38	0.41	0.61	0.66
		Nonlinear optimization solution	0.25	0.33	0.49	0.54
	30 mm	Close form solution	0.52	0.56	0.69	0.89
		Nonlinear optimization solution	0.36	0.44	0.64	0.74
	40 mm	Close form solution	0.67	0.76	1.02	1.15
		Nonlinear optimization solution	0.46	0.66	0.83	0.94
4	10 mm	Close form solution	0.25	0.30	0.41	0.47
		Nonlinear optimization solution	0.18	0.27	0.35	0.42
	20 mm	Close form solution	0.42	0.47	0.66	0.73
		Nonlinear optimization solution	0.28	0.41	0.54	0.60
	30 mm	Close form solution	0.57	0.63	0.77	0.98
		Nonlinear optimization solution	0.41	0.58	0.72	0.83
	40 mm	Close form solution	0.74	0.87	1.11	1.27
		Nonlinear optimization solution	0.53	0.76	0.94	1.06

**Figure 7:**
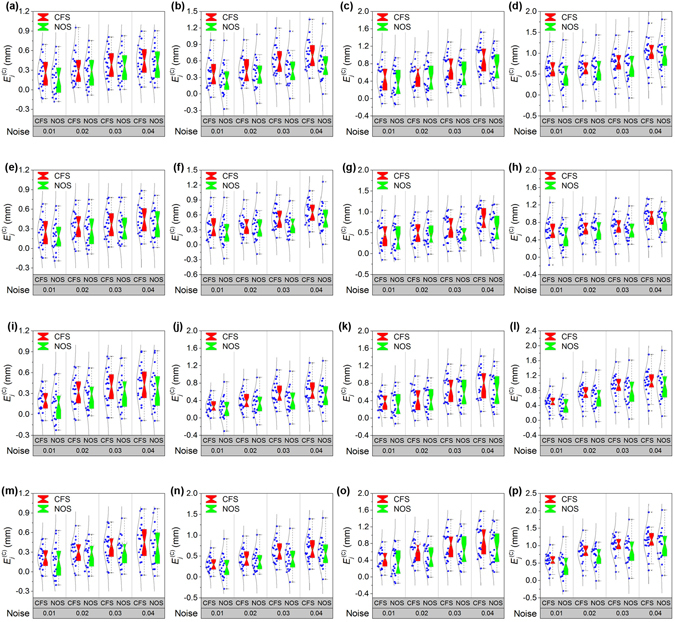
Experimental results of the close form solution (CFS) and the nonlinear optimization solution (NOS) with respect to the lens distortion. The noise levels are 0.01, 0.02, 0.03 and 0.04, respectively. (**a**–**d**) The first group of comparison results of CFS and NOS in the displacements of 10 mm, 20 mm, 30 mm and 40 mm, respectively. (**e**–**h**) The second group of comparison results of CFS and NOS in the displacements of 10 mm, 20 mm, 30 mm and 40 mm, respectively. (**i**–**l**) The third group of comparison results of CFS and NOS in the displacements of 10 mm, 20 mm, 30 mm and 40 mm, respectively. (**m**–**p**) The fourth group of comparison results of CFS and NOS in the displacements of 10 mm, 20 mm, 30 mm and 40 mm, respectively.

In the 3D reconstruction process of the displacement between the laser points, the lens distortion is the other important factor to be evaluated. The effects of the lens distortion on the displacement reconstructions are investigated in the experiments. The experimental results of NOS and CFS with regard to the distortion effects are shown in Fig. 7. Four groups of experiments with regard to the lens distortion are performed to compare the effects of the noises and displacements. The statistical results are listed in Table 2. In the experiments above, the results reflect that the error variances of CFS and NOS show the growing trend with the increasing noise level. The means of errors based on CFS and NOS rise as the displacement increases. The error variations of NOS method are closer to the real displacements than CFS method under the identical noise and displacement. The errors prove that NOS provides high accuracy for the 3D reconstructions of the laser projective points.

**Table 2:** The error means of the close form solution (CFS) and the nonlinear optimization solution (NOS) with regard to the lens distortion related to Fig. 7.

Group	Displacement	Solution method	Error mean			
			Noise 0.01	Noise 0.02	Noise 0.03	Noise 0.04
1	10 mm	Close form solution	0.25	0.33	0.38	0.45
		Nonlinear optimization solution	0.17	0.28	0.36	0.41
	20 mm	Close form solution	0.37	0.42	0.61	0.71
		Nonlinear optimization solution	0.27	0.37	0.45	0.53
	30 mm	Close form solution	0.45	0.51	0.69	0.91
		Nonlinear optimization solution	0.38	0.48	0.57	0.75
	40 mm	Close form solution	0.58	0.62	0.75	1.00
		Nonlinear optimization solution	0.48	0.59	0.69	0.95
2	10 mm	Close form solution	0.25	0.34	0.38	0.46
		Nonlinear optimization solution	0.18	0.28	0.34	0.38
	20 mm	Close form solution	0.36	0.39	0.54	0.66
		Nonlinear optimization solution	0.26	0.35	0.41	0.54
	30 mm	Close form solution	0.44	0.50	0.60	0.84
		Nonlinear optimization solution	0.37	0.47	0.49	0.63
	40 mm	Close form solution	0.56	0.64	0.70	0.91
		Nonlinear optimization solution	0.47	0.62	0.65	0.83
3	10 mm	Close form solution	0.19	0.31	0.39	0.42
		Nonlinear optimization solution	0.14	0.26	0.34	0.37
	20 mm	Close form solution	0.27	0.39	0.56	0.61
		Nonlinear optimization solution	0.22	0.33	0.41	0.53
	30 mm	Close form solution	0.38	0.45	0.67	0.79
		Nonlinear optimization solution	0.32	0.42	0.60	0.64
	40 mm	Close form solution	0.49	0.71	0.91	1.01
		Nonlinear optimization solution	0.41	0.62	0.78	0.93
4	10 mm	Close form solution	0.21	0.30	0.39	0.45
		Nonlinear optimization solution	0.16	0.26	0.32	0.39
	20 mm	Close form solution	0.31	0.44	0.62	0.68
		Nonlinear optimization solution	0.25	0.38	0.45	0.57
	30 mm	Close form solution	0.44	0.61	0.75	0.88
		Nonlinear optimization solution	0.35	0.47	0.66	0.71
	40 mm	Close form solution	0.56	0.81	0.99	1.13
		Nonlinear optimization solution	0.43	0.70	0.86	1.02

Taking all the factors into consideration, we get the conclusion that the average errors of CFS and NOS grow with the increase of noise. Moreover, the reconstruction results of NOS considering the lens distortion contribute a higher accuracy and are closer to the true values. In addition, the average errors of both CFS and NOS decline with the decreasing displacement.

**Figure 8:**
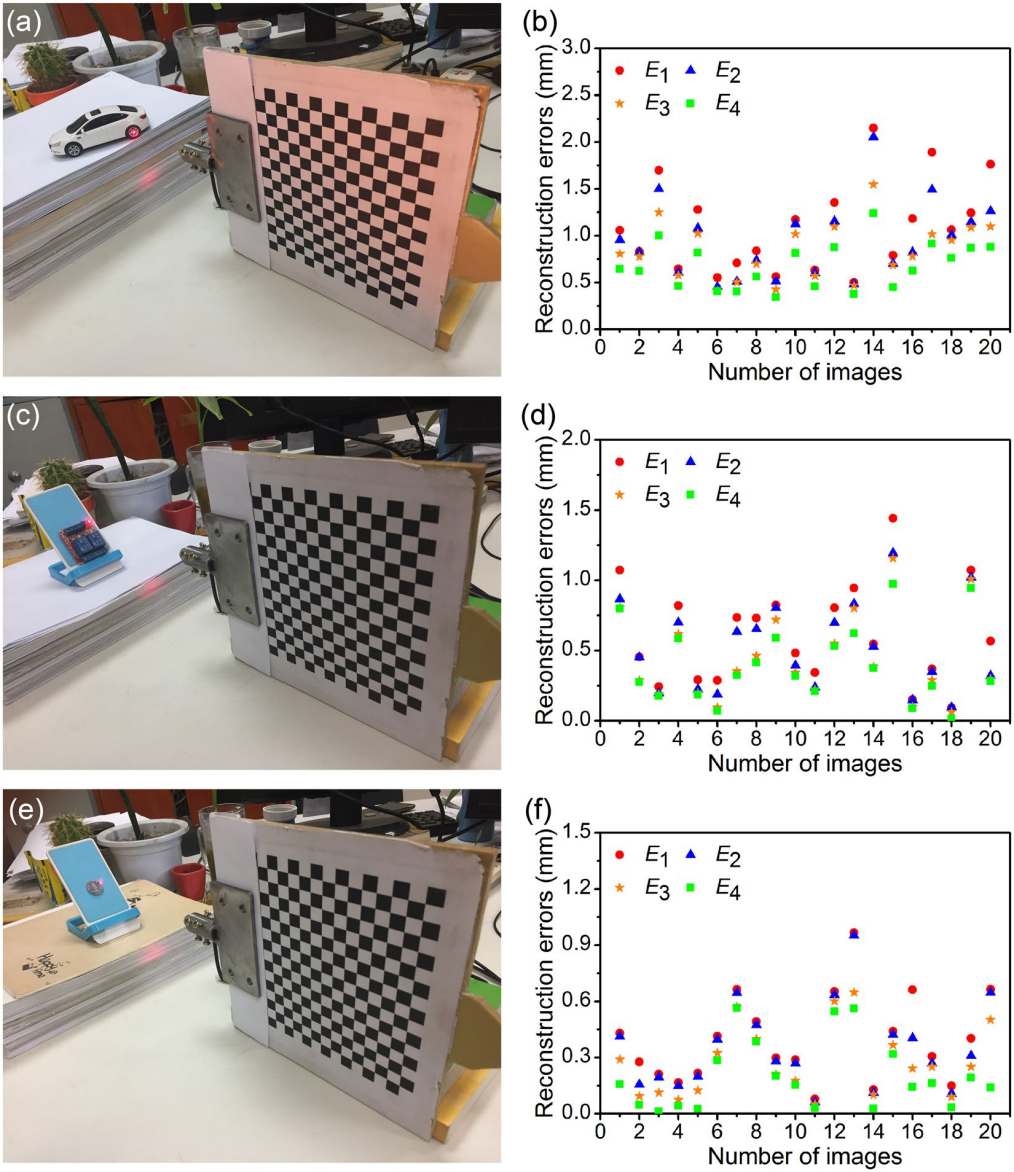
Three examples of the applications using the close form solution (CFS) and the nonlinear optimization solution (NOS). The first example is to measure the wheelbase of a car model. The second example is to test the distance between two positioning holes on a circuit board. The third one is to obtain the diameter of a coin. *E*_1_ is the reconstruction error of CFS without respect to the lens distortion. *E*_2_ is the reconstruction error of CFS with respect to the lens distortion. *E*_3_ is the reconstruction error of NOS without respect to the lens distortion. *E*_4_ is the reconstruction error of NOS with respect to the lens distortion. (**a**) The measurement photo of the first example. (**b**) The measurement results of the first example. (**c**) The measurement photo of the second example. (**d**) The measurement results of the second example. (**e**) The measurement photo of the third example. (**f**) The measurement results of the third example.

Three application cases are provided to further show the applications of the reconstruction method. The wheelbase of a car model, the distance between two holes on the circuit board and the diameter of a coin are chosen as the objects to be measured. Every measured value is tested by the reconstruction method twenty times. The benchmarks of the tests are determined by the measurement outputs of a vernier caliper. The benchmark values are 64.00 mm, 41.07 mm and 19.08 mm, respectively. The measurement results are shown in Fig. 8. Four kinds of the reconstruction errors are considered in the three cases. The means of the reconstruction errors, CFS without respect to the lens distortion, are 1.10 mm, 0.61 mm and 0.40 mm in the three measurements. Then, the means of the reconstruction errors, CFS with respect to the lens distortion, are 0.95 mm, 0.53 mm and 0.35 mm in the three measurements. Furthermore, the means of the reconstruction errors, NOS without respect to the lens distortion, are 0.84 mm, 0.45 mm and 0.27 mm in the three measurements. However, the means of the reconstruction errors, NOS with respect to the lens distortion, are 0.68 mm, 0.40 mm and 0.20 mm, respectively. The NOS with respect to the lens distortion demostrates the best performance in the three applications. Its average error is 0.43 mm, which proves that the reconstruction method can be used in some non-contact measurements and inspections.

## Discussion

A method to reconstruct the 3D laser point is proposed in the paper and realized by the project invariant generated from five points on a target plane. A laser projector is fixed on the 2D target in order to locate the laser point and the points of the 2D target on the same plane. The projective invariants are obtained from the laser projective point and the five non-collinear points that are randomly selected from the 2D target. The coordinates of the laser points are initially determined by the singular value decomposition. The optimization function is constructed by the differences between the re-projection points and the ideal points of the 2D target points and the laser points that are extracted in the image coordinate system. The 3D reconstructions of the laser projective points are finally obtained by minimizing the parameterized re-projection errors in the optimization function. The effects of the image quantity, the lens distortion and the displacement are analyzed by the experiments. In the first group of experiments, the error means are 0.30 mm, 0.40 mm, 0.54 mm and 0.68 mm on the condition that the displacement grows from 10 mm to 40 mm. In the second group of experiments, the error means are 0.29 mm, 0.39 mm, 0.49 mm and 0.64 mm. In the third group of experiments, the error means are 0.28 mm, 0.37 mm, 0.49 mm and 0.69 mm. In the fourth group of experiments, the error means are 0.28 mm, 0.41 mm, 0.55 mm and 0.75 mm. The error mean is 0.47 mm in the four groups of experiments. Considering the reasonable measurement scope in the experiments, it is necessary to evaluate the relative errors of the reconstruction method in the experiments. The relative errors of the first group are 3.03%, 2.02%, 1.82% and 1.69%. The relative errors of the second group are 2.94%, 1.96%, 1.63% and 1.61%. The relative errors of the third group are 2.77%, 1.86%, 1.65% and 1.72%. The relative errors of the fourth group are 2.82%, 2.06%, 1.82% and 1.88%. The largest relative error is 3.03%, which is less than 5% of the normal measurement instruments, in the four groups of experiments. The error variation grows with the increasing displacement. The projection invariant realizes the 3D reconstructions of the laser projective points. Moreover, the optimization function considering the lens distortion effectively promotes the reconstruction accuracy of the 3D laser projective points, which has the applicable potential to detect and reconstruct the 3D feature points or profile of objects.

## Summary

In summary, a reconstruction method for a laser projective point is discussed by the invariant determined by the five points on a 2D target. The close form solution is achieved by the differences between the image-coordinate-invariants and world-coordinate-invariants. The nonlinear solution is conducted by minimizing the parameterized re-projection errors. The experiment results verify that the mean of the measurement errors is 0.47 mm and the largest measurement error is 0.75 mm. The mean of the relative errors is 2.08% and the largest relative error is 3.03%. It is proved that the reconstruction method can support the applications in more general systems of the non-contact measurements and inspections. In future works, the improvement method to enlarge the measurement scope should be investigated for the wide applications.
